# Lockdowns and their influence on Earth’s hum

**DOI:** 10.1038/s41598-021-97459-1

**Published:** 2021-09-08

**Authors:** Surendra Nadh Somala

**Affiliations:** Indian Institute of Technology Hyderabad, Hyderabad, India

**Keywords:** Environmental sciences, Natural hazards

## Abstract

Earth’s hum at higher frequencies is disturbed substantially by human activity. Anthropogenic noise is more evident in frequencies higher than 1 Hz. The power at 10 Hz power is used from January 2020 to early May (mostly first wave of SARS-CoV-2) across various sites across the world, to show that there is a clear decrease in noise power during the lockdown period. Furthermore, this anthropogenic noise across the world during the COVID-19 lockdown period, within which vehicular movement and industrial activity have stalled in many places, is quantified into a few bins. Implications of easing the lockdown measures on the onset of second wave of pandemic are discussed.

## Introduction

The COVID-19 crisis is coming in multiple waves and is impacting different countries with varying timeline. Some of the countries are into the third while a few others are inching into the fourth wave of the pandemic. The authorities of most of the countries have imposed lockdowns to stop the spread of virus. These lockdowns were mostly imposed nationwide during the first wave but were largely restricted to small regions during the second and further waves of SARS-CoV-2. Lockdowns have indirectly contributed to restoring the Earth’s hum at almost all the frequency bands and shed light on the lowest plausible seismic noise at each place when anthropogenic activity is at its minimum.

Anthropogenic noise recorded by ground-based vibration is dominant in higher frequencies^1,2^. The main contributing factor of this anthropogenic noise is traffic, including noise from the trains^3^. Another important source is the machinery operated in the energy sector^4^. Diurnal and seasonal patterns of human-induced vibrations have been studied in the literature^5^. The novelty of this study is to quantify how lockdowns have influenced the noise power in the anthropogenic frequency band across the entire world using publicly available ground vibration data and to see if there is any correlation between number of daily new cases reported by the World Health Organization (WHO) and increase in anthropogenic noise beyond the first wave of SARS-CoV-2.

## Data and resources

**Figure 1:**
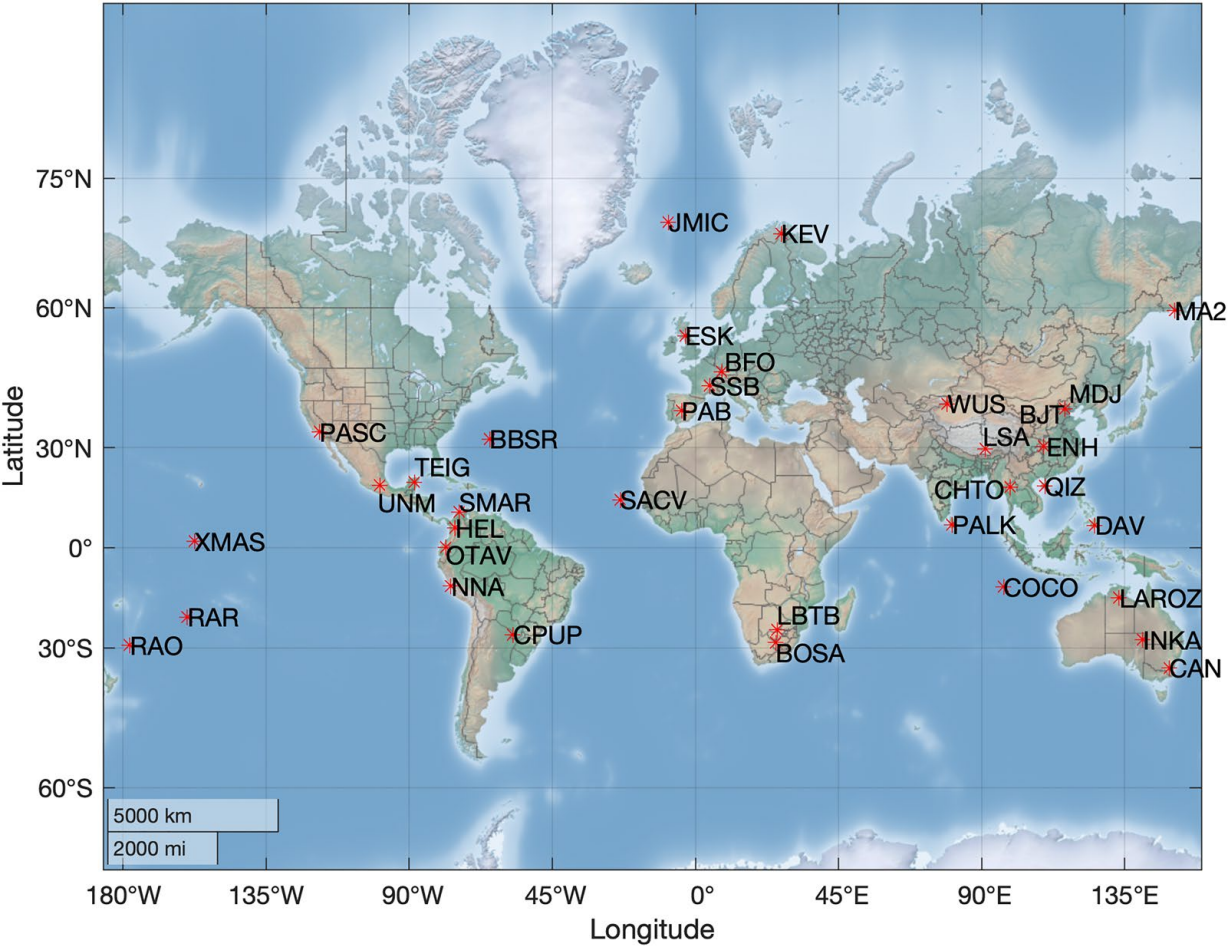
Locations across the world where anthropogenic noise reduction is observed during January to early May 2020. Matlab version 2020a was used to make this map (https://matlab.mathworks.com/).

All the seismic stations from the Federation of Digital Seismograph Network (FDSN) are used to examine the ambient noise power at 10 Hz, downloadable from Incorporated Research Institutions for Seismology (IRIS). Out of all those station seismic stations in FDSN, the locations where there is a clear decrease in noise power are shown in Fig. 1. The red stars in Fig. 1 are spread all over the world map indicating the extent of impact the COVID-19 crisis had on the planet Earth. Large regions without stations in Fig. 1 does not necessarily imply a lack of reduction in noise power but there is a possibility of data not being freely available. Nevertheless, the available locations cover almost all the continents along with a few stations in the islands.

## Methodology

The power of the ground acceleration signal is computed relative to 1 m^2^/s^4^/Hz^6^ and is expressed in terms of Decibels (dB) as shown in Eq. (1).

1 $${\text{P}}\left[dB\right]=10{\mathrm{log}}_{10}\left[P / 1 {\left(m/{s}^{2}\right)}^{2}/{\text{Hz}}\right]$$

The power can be evaluated any frequency of interest. In this work, to understand the variations within the anthropogenic band, power at 10 Hz is considered throughout this study. The power at 10 Hz is computed for each and every day throughout the several months considered in this study. The initial part is focussed on early part of 2020 during the first wave of COVID-19 to quantify the reduction in anthropogenic noise. The latter part of the covers almost one and half year from Jan 2020 to July 2021 to understand the correlation with daily new cases reported by WHO.

## Results and discussion

**Figure 2:**
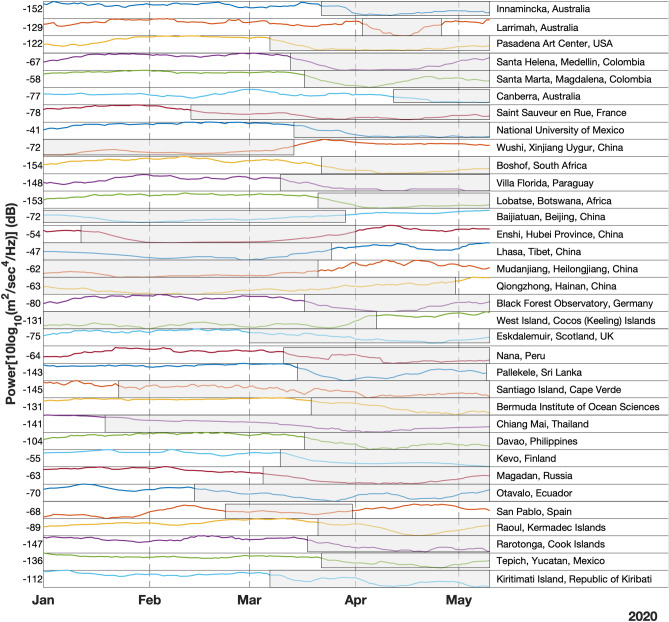
Seismic noise power in dB, highlighting time-period during which anthropogenic noise is lower. The text to the right of each curve represents the network and station codes. The grey shading represents mostly the lockdown periods pertinent to those sites. The value to the left of each curve is the mean of upper and lower limits of noise during the time period considered (January to early May 2020).

The moving-mean over 15 days of noise power relative to 1 m^2^/s^4^/Hz^6^ is shown in Fig. 2 at the locations indicated by red stars in Fig. 1. It can be observed that for stations with network code IC the lower levels of noise power has started in the month of January itself. These are stations located in China where the COVID-19 crisis was realised in December 2019 and lockdown measures have started by January 2020. At these stations, anthropogenic noise started picking up by mid to late March 2020 possibly implying lifting of lockdown measures. The WUS station of Geoscope (G) network, which shows lower noise during January and February, is also located in China. The majority of other locations have shown a greater reduction in noise during March to April time-frame.

The highest contribution to anthropogenic noise is observed in Nana of Peru (NNA), and Innamincka of Australia (INKA) to be about 18 dB, followed by Bermuda (BBSR), and Santiago Island of Cape Verde (SACV) showing close to 15 dB reduction. This reduction is in comparison to the overall average of minimum and maximum power recorded for the months considered in this study.

Among the locations that showed 11–15 dB anthropogenic noise levels are Chiang Mai of Thailand (CHTO), Enshi in Hubei of China (ENH), Qiongzhong in Hainan of China (QIZ), Larrimah in Northern Territory of Australia (LAROZ), and Lhasa of Tibet (LSA). There are also a good number of locations showing a reduction in anthropogenic noise close to 10 dB like the Pasadena Arts College (PASC), Villa Florida of Paraguay (CPUP), Cocos (Keeling) Islands (COCO), and Wushi in Xinjiang Uygur of China (WUS).

Finally, locations with considerable (> 5 dB) but less than 10 dB anthropogenic noise are Kiritimati Island (XMAS), Rarotonga of Cook Islands (RAR), Tepich in Yucatan of Mexico (TEIG), Kevo of Finland, Otavalo of Ecuador (OTAV), Santa Helena in Medellin of Colombia (HEL), Pallekele of Sri Lanka (PALK), Davao of Philippines (DAV), Eskdalemuir of Scotland (ESK), Raoul of Kermadec Islands (RAO), and Canberra of Australia (CAN).

Rest of the locations that are shown in Fig. 1 but have not been mentioned thus far have shown < 5 dB reduction in noise from anthropogenic sources. A few such locations that are worth mentioning are Lobatse in Botswana of Africa (LBTB), Mudanjiang in Heilongjiang Province of China (MDJ), Black Forest Observatory in Schiltach of Germany (BFO), San Pablo of Spain (PAB), Saint Sauveur en Rue of France (SSB), Magadan of Russia (MA2), Beijing of China (BJT), and Boshof of South Africa (BOSA).

**Figure 3:**
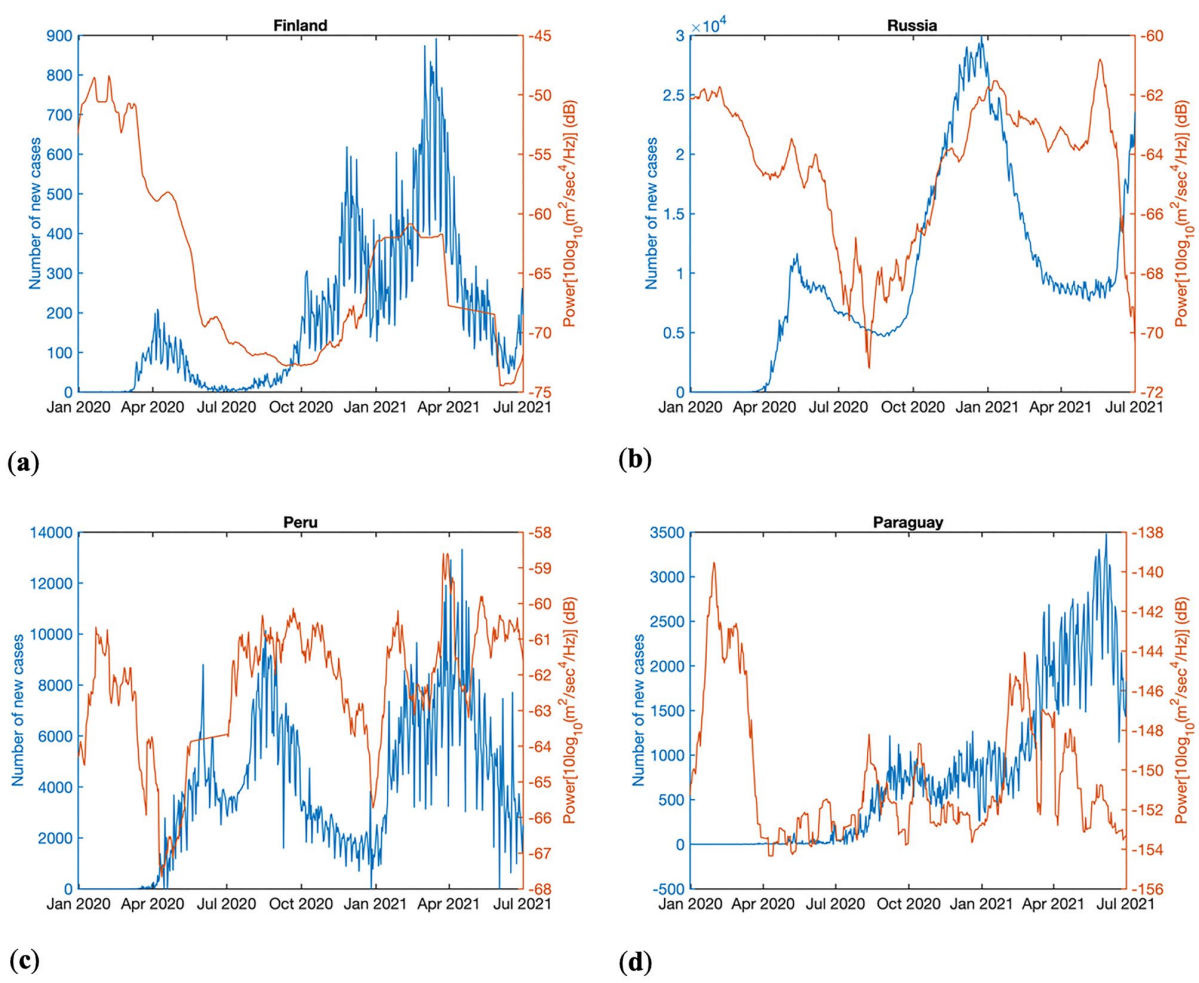
Correlation between second wave of peak in new cases and increasing in seismic noise (easing of lockdowns) for (**a**) Finland (**b**) Russia (**c**) Peru (**d**) Paraguay.

Figure 3 shows the number of daily new cases reported by WHO together with the anthropogenic noise power for four different countries—namely, Finland, Russia, Peru and Paraguay. Each of these countries broadly correspond to one each from each of the bins of noise reduction identified during the lockdowns. It can be seen from Fig. 3 that after the initial reduction in anthropogenic ground noise due to lockdowns, the increase beyond a local minimum could be attributed to the easing of lockdown measures. Such an increase is seen to occur within the year 2020 itself, when vaccines were still not readily available to public. The secondary peak of number of cases does appear to align well with the easing of lockdowns. Anthropogenic noise monitoring can thus provide precursory indicators for secondary and further waves of pandemics.

## Conclusion

The reduction in anthropogenic noise during the first few months of 2020 is attributed to the lockdown measures that were implemented in various countries due to the prevalent COVID-19 crisis. Under this assumption, the noise power at 10 Hz is evaluated at locations where seismic data access is available through IRIS and classified them into categories of high (> 10 dB), medium (5–10 dB) and low (< 5 dB) cultural noise sites. While this classification also has to do with how strictly lockdown measures are implemented in that particular location, this unprecedented crisis is almost like a once in a lifetime extreme event that it is worth documenting the reduction in ambient noise powers.

The present study finds that the anthropogenic noise is as high as 18 dB. This is not necessarily an upper bound as not all the FDSN stations are accessible. Islands fall mostly in the medium to the high category of anthropogenic noise. Finally, easing of lockdown restrictions after the first wave of SARS-CoV-2 has shown clear increase in anthropogenic noise, which appear to correlate well with the secondary peak of daily cases related to the second wave. Limitations of this study include restricting it to just one of the frequencies. Moreover, a single station being representative of a nation is an extrapolation made to overcome the limited available of ground vibration data. Extensive seismic monitoring has potential to serve as “early warning” for secondary waves of pandemics. Hence, a more organized seismological monitoring with advanced technologies, within the purview of the United Nations, can help achieve one of its sustainable developments goals of overseen by the United Nations Office for Disaster Risk Reduction.

## Data Availability

All data is freely available to anyone through Incorporated Research Institutions for Seismology (https://www.iris.edu/hq/). WHO data on daily cases is also publicly accessible on https://covid19.who.int/info/ (last accessed: 6th August, 2021).
